# Dysfunction in dynamic, but not static balance is associated with risk of accidental falls in hemodialysis patients: a prospective cohort study

**DOI:** 10.1186/s12882-022-02877-6

**Published:** 2022-07-06

**Authors:** Nobuyuki Shirai, Suguru Yamamoto, Yutaka Osawa, Atsuhiro Tsubaki, Shinichiro Morishita, Ichiei Narita

**Affiliations:** 1 Department of Rehabilitation, Niigata Rinko Hospital, Niigata, Japan; 2 Division of Clinical Nephrology and Rheumatology, Niigata University Graduate School of Medical and Dental Sciences, 1-757 Asahimachi-dori, Niigata, 951-8510 Japan; 3 Internal Medicine, Niigata Rinko Hospital, Niigata, Japan; 4 Institute for Human Movement and Medical Sciences, Niigata University of Health and Welfare, Niigata, Japan; 5 Department of Physical Therapy, School of Health Science, Fukushima Medical University, Fukushima, Japan

**Keywords:** Accidental falls, Dynamic balance, Static balance, Hemodialysis

## Abstract

**Background:**

Patients with chronic kidney disease undergoing hemodialysis (HD) have a high incidence of falls. Impairment of balance function is a risk factor for falls in the general elderly, and no report examining the association between balance dysfunction and fall incidence in HD patients exists.

**Methods:**

This prospective cohort study was conducted at a single center. The timed-up-and-go test (TUG) as a dynamic balance function was performed and length of the center of pressure (CoP) as a static balance function was measured before and after the HD session at baseline. Data of the number and detailed information of accidental falls for 1 year were collected. Multiple regression analyses were performed to assess the relationships between the number of falls and balance function.

**Results:**

Forty-three patients undergoing HD were enrolled in the study. During 1 year of observation, 24 (55.8%) patients experienced accidental falls. TUG time was longer, and CoP was shorter in the post-HD session than in the pre-HD session. Adjusted multiple regression analyses showed that the number of accidental falls was independently associated with TUG time in the pre-HD session (B 0.267, *p* < 0.001, R^2^ 0.413) and that in the post-HD session (B 0.257, *p* < 0.001, R^2^ 0.530), but not with CoP.

**Conclusions:**

Dynamic balance was associated with fall incidence in maintenance HD patients. The evaluation and intervention of dynamic balance function might reduce the risk of falls in HD patients.

**Trial registration:**

This study was carried out with the approval of the Niigata Rinko Hospital Ethics Committee (approval number 2005–92) (Registered on December 11, 2019) and registered in The University Hospital Medical Information Network (registration number 000040618).

**Supplementary Information:**

The online version contains supplementary material available at 10.1186/s12882-022-02877-6.

## Background

Patients with chronic kidney disease (CKD), especially those undergoing hemodialysis (HD), present a high frequency of falls and fractures [1, 2]. Several dialysis-related factors, including sarcopenia [3] and fluid volume change with HD treatment [4], are thought to be associated with fall incidence, whereas the direct association with physical functions is not fully understood.

Balance dysfunction is commonly cited as a risk factor for falls in the general population [5, 6]. Dynamic balance is an indicator of walking ability, whereas athletic ability is required for performing basic activities in daily life [7]. Static balance is an index of postural control and postural stability [8]. Balance function may be impaired in HD patients with a high risk of falls and fractures; however, to the best of our knowledge, studies investigating the association of dynamic or static balance with fall incidence in HD patients have not been reported to date. This prospective observational study evaluated the details of accidental falls and their association with balance function in HD patients.

## Methods

### Study design and participants

This prospective observational study was conducted in a single hospital. All HD patients at Niigata Rinko Hospital from December 2019 to May 2020 were recruited. The eligibility criteria were patients aged ≥20 years who had received HD for ≥3 months and consented to participate in this study. Patients in the hospital complaining of severe hypotension during or after HD and difficulty walking were excluded from the study. Patients who died or were transferred were excluded. Patient information was collected from medical records. Age, sex, height, dry weight (DW), change of DW in a year (%), body mass index, dialysis duration, primary cause of CKD, presence of comorbidities (including cerebrovascular disease, cardiovascular disease, diabetes, and diabetic retinopathy), Charlson Comorbidity Index (CCI), serum albumin level, blood pressure before and after dialysis, single pool Kt/v for urea, water removal amount, number of medications, blood hemoglobin level, and serum intact parathyroid hormone level, Japan Cardiovascular Health Study (J-CHS) and history of fall during a year were reported as basic characteristics. The balance functions before and after the HD session were measured by a physiotherapist.

### Evaluation of accidental falls

An accidental fall was defined as an unexpected event in which the participants rested on the ground, floor, or lower level [9]. Details of accidental falls, including timing, location, and action [10], were reported every 2 weeks. Falls that required medical care or hospitalization were defined as severe falls [11].

### Dynamic balance evaluation

The timed-up-and-go test (TUG) evaluates dynamic balance. Patients were seated in a chair with a backrest and placed the soles of their feet on the ground. Then, they stood up at the start signal, walked to the cone 3 m away at a maximum walking speed, turned around the cone, and were seated in the chair again. This event was performed twice. The time was measured, and the faster one was adopted [7].

### Static balance evaluation

Body sway was measured using a force platform (Win-Pod manufactured by Medicapteurs, France). The length of the center of pressure (CoP), in the open-eye standing position, was evaluated to assess static balance. The length of CoP was the distance traveled by the foot CoP. The posture was a closed leg standing position, with both arms alongside the body and the bare feet placed on the platform plate. Data were measured once at a sampling rate of 100 Hz. The patient had a black circle set 250 cm apart and maintained a constant posture for 60 s [12].

### Statistical analysis

Each indicator is shown as median (interquartile range) and percentage (%). The participants were divided into two groups by Pre-HD TUG consisting of the faster group and the slower group, and background data were compared using Mann-Whitney U test and chi-square test. The Spearman’s rank correlation coefficient test was performed to investigate the correlation between the number of falls and balance function before and after HD. In the multiple regression analyses, the number of falls and balance function were set as the independent and dependent variables, respectively. The covariates included age, sex and CCI [10, 13] for the main analysis, and age, history of falls and number of medications for another analysis. The Wilcoxon signed-rank test was performed to compare the dynamic and static balance before and after dialysis. IBM SPSS Statistics version 27 was used for statistical analyses, and the significance level was set at 5% in each case.

## Results

**Fig. 1:**
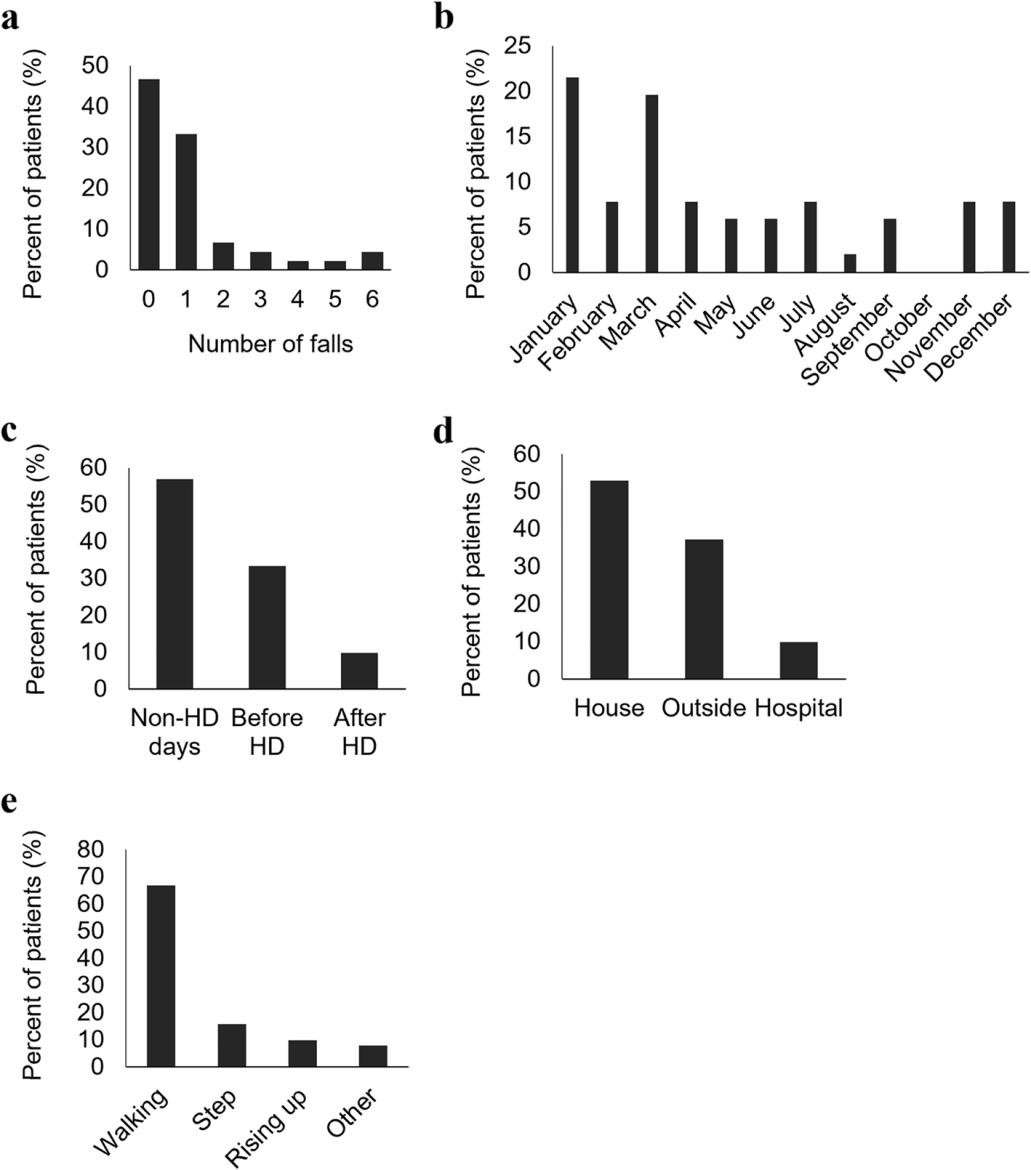
Details of accidental falls in patients undergoing hemodialysis (HD). Frequency of accidental falls in **a** year, **b** season, **c** timing, **d** location, and **e** action are shown

This study analyzed 43 patients with HD. Table 1 presents the clinical characteristics of the participants. The median age of the participants was 74.0 (66.0–79.0) years. The study included 20 (46.5%) men, and the median dialysis period was 5.0 (2.0–11.0) years. Median TUG was 8.9 (7.6–10.6) s at Pre-HD TUG. The slower TUG group showed older, higher J-CHS score, taking much number of medications, and frequent history of falls. (Table 1) Fig. 1a shows the frequency of falls within 1 year. Out of 51 accidental falls (1.19/person/year), 24 (55.8%) patients experienced accidental falls once and 9 (37.5%) patients experienced accidental falls at least twice. Severe falls occurred 17 (33.3%) times, and fractures associated with falls occurred 8 (15.7%) times. The frequency of falls was high in January and March (Fig. 1b) and while walking outside. There were more accidental falls on non-HD days than on HD days (Fig. 1c). On HD days, the incidence of falls was higher before HD than after HD and occurred most frequently in the house (52.9%) (Fig. 1d) and while walking (66.7%) (Fig. 1e). Median length of CoP was 2165.9 (1756.8–2670.3) mm at Pre-HD (Supplement Table 1). No significant difference was found between the shorter and the longer groups.

**Fig. 2:**
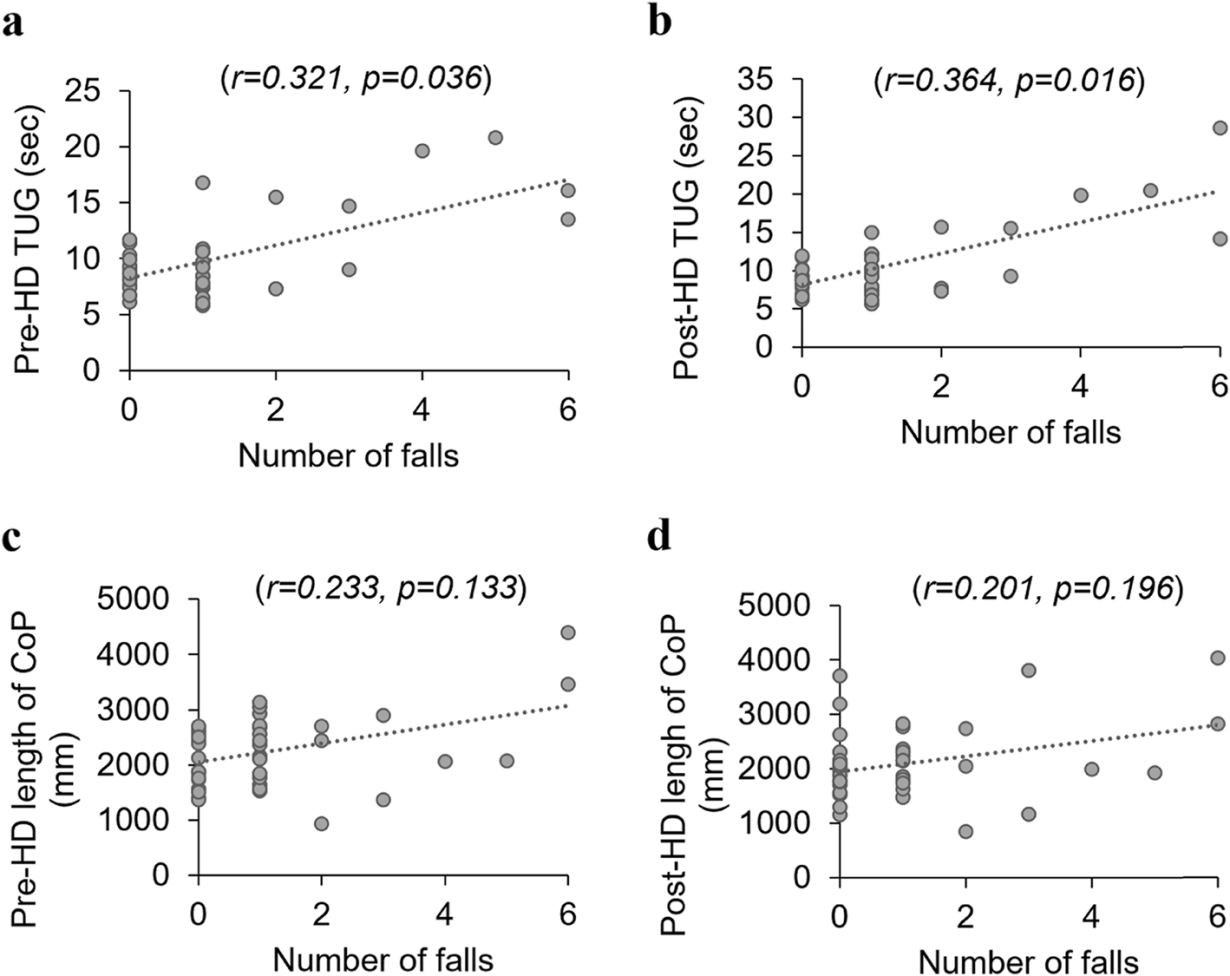
Correlation between the frequency of falls and balance functions. Correlation of frequency of accidental falls with timed-up-and-go test (TUG) at pre- **a** and post- **b** hemodialysis (HD) treatment, and length of center of pressure (CoP) at pre- **c** and post- **d** HD treatment are shown

TUG, an index of dynamic balance, at the post-HD session was slower than that at the pre-HD session [9.3 (7.8–11.8) vs. 8.9 (7.6–10.6) s, *p = 0.002*]. The length of CoP, an index of static balance, at pre-HD was longer than that at post-HD [2165.9 (1762.2–2670.3) vs. 1992.2 (1703.0–2315.1) mm, *p = 0.020*] (Additional file 1). Figure 2 shows the association between the number of accidental falls and balance functions before and after HD. A positive correlation was found between the number of falls and TUG time, both at pre-HD and post-HD sessions (pre-HD: *r 0.321, p = 0.036*; post-HD: *r 0.364, p = 0.016*). However, no correlation was found between the length of CoP and the number of accidental falls (pre-HD: r 0.233, *p* = 0.133, post-HD: r 0.201, *p* = 0.196). TUG time at the pre-HD session (B 0.267, *p* < 0.001, R^2^ 0.413) and post-HD session (B 0.257, *p* < 0.001, R^2^ 0.530), but not CoP, were independently associated with the number of accidental falls in HD patients in the multiple regression analysis adjusted with age, sex and CCI (Table 2). Same trend was found when we used covariates including age, history of falls and number of medications (Supplement Table 2).

**Table 2:** Relationship between the number of falls and balance functions

	Unadjusted model		Adjusted model^*^	
	B (95% CI)	*p*-value	B (95% CI)	*p*-value
Pre-HD TUG	0.297 (0.190, 0.404)	< 0.001	0.267 (0.148, 0.385)	< 0.001^a^
Post-HD TUG	0.266 (0.189, 0.344)	< 0.001	0.257 (0.169, 0.344)	< 0.001^b^
Pre-HD length of CoP	0.001 (0.000, 0.002)	0.006	0.001 (0.000, 0.001)	0.064^c^
Post-HD length of CoP	0.001 (0.000, 0.001)	0.033	0.001 (0.000, 0.001)	0.114^d^

*CI* Confidence Interval, *CoP* Center of Pressure, *TUG* Timed-up-and-go test.

^*^Adjusted model: Age, sex, Charlson Comorbidity Index

^a^Adjusted R2 = 0.413, ^b^adjusted R2 = 0.530, ^c^adjusted R2 = 0.172, and ^d^adjusted R2 = 0.151

## Discussion

This study reported the relationship between accidental falls and balance functions in patients undergoing HD treatment. The main result of this study indicated that 55.8% of HD patients experienced falls within 1 year, and TUG was associated with the frequency of falls.

It has been reported that approximately 30% of community-dwelling elderly, aged ≥70 years, experience a yearly fall [14]. In this study, accidental falls occurred in 55.8% of HD patients whose median age was 74.0 years (Fig. 1a), suggesting that the frequency of falls in HD patients was much higher than in the general elderly population, as previously reported [10, 15, 16]. Frequent falls more than twice a year and fractures associated with falls occurred in 33.3 and 15.7% of patients, respectively. Therefore, it would be necessary to formulate a fall prevention program for HD patients to avoid fracture incidence. Most falls occurring in January and March while walking outside (Fig. 1b) was possibly due to snowfall and ice in winter [17]. Accidental falls frequently occurred on non-dialysis days and before HD sessions (Fig. 1c). Non CKD-elderly men when they are active with low physical function experience frequent accidental falls [18]. A large-scale study investigating the amount of physical activity in HD patients has reported 2912 steps on HD days and 4054 steps on non-HD days [19]. Thus, HD patients with impaired physical function might have more chances of accidental falls on non-HD days. Although a previous study has shown that more falls occurred on post-HD session than on pre-HD [10], our study included the elderly population and had impaired physical function and fatigue after HD sessions, plausibly limiting physical activity.

Factors generally affecting balance function include the visual, vestibular, somatosensory, musculoskeletal, and central nervous systems [20]. In this study, the TUG slower group was older than the TUG faster group. (Table 1) We examined the multiple analysis was adjusted with age, but it may be affected by unmeasured confounders related with age in the association between balance functions and fall incidence in HD patients as same as the general population [21]. The number of falls was correlated with the TUG test in HD patients (Fig. 2 and Table 2), possibly due to impaired lower limb muscle strength in patients undergoing HD [22]. Increased proteolysis [23], accumulation of uremic toxins [24, 25], and vitamin D deficiency [26] might be associated with muscle mass loss, which might further be related to impaired dynamic balance function in HD patients. However, no relationship between static balance and the risk of falls was observed (Fig. 2 and Table 2). A previous study has suggested that a higher CoP range with the open-eye standing position is not associated with the odds of falling in HD patients [27]. Although the lower limb muscle strength, the main component of dynamic balance, can affect falls [28], static balance is affected by vision [29] and somatosensory [30] rather than muscle strength. Therefore, the static balance was considered to have no clear association with fall incidence in HD patients in our study.

**Table 1:** Participants’ clinical characteristics and comparison by pre-HD TUG

Parameters	All (***n*** = 43)	TUG faster group (***n*** = 22)	TUG slower group (***n*** = 21)	***p***-value
Age (years)	74.0 (66.0–79.0)	70.0 (64.8–78.3)	76.0 (70.0–82.5)	0.005
Men, n (%)	20 (46.5)	11 (50.0)	9 (42.9)	0.763
Height (m)	1.56 (1.47–1.63)	1.56 (1.47–1.66)	1.53 (1.47–1.63)	0.106
DW (kg)	51.0 (42.8–60.6)	55.6 (43.3–60.8)	47.0 (42.7–60.6)	0.068
Change of DW (%)	0.0 (−5.0–3.0)	0.0 (−1.4–3.5)	−2.2 (−8.0–1.1)	0.089
BMI (DW/m^2^)	20.6 (19.0–23.4)	21.1 (18.3–23.4)	20.6 (18.7–23.0)	0.308
Dialysis duration (years)	5.0 (2.0–11.0)	4.5 (2.0–11.3)	5.0 (2.0–11.0)	0.441
Comorbid conditions				
Cerebrovascular disease, n (%)	11 (25.6)	6 (27.2)	5 (23.8)	1.0
Cardiac disease, n (%)	28 (65.1)	16 (72.7)	12 (57.1)	0.347
Diabetes mellitus, n (%)	19 (44.1)	11 (50.0)	8 (38.1)	0.543
Diabetic retinopathy, n (%)	9 (20.9)	6 (27.3)	3 (14.3)	0.457
Primary kidney disease				
Diabetic nephropathy, n (%)	11 (25.6)	5 (22.7)	6 (28.6)	0.736
Glomerulonephritis, n (%)	14 (32.6)	7 (31.8)	7 (33.3)	1.0
Hypertension, n (%)	11 (25.6)	6 (27.3)	5 (23.8)	1.0
Other nephropathies, n (%)	7 (16.3)	4 (18.2)	3 (14.3)	1.0
CCI (score)	6.0 (4.0–7.0)	5.5 (4.0–7.0)	7.0 (5.0–7.0)	0.843
Blood pressure				
Before dialysis SBP (mmHg)	154.0 (141.0–172.6)	153.0 (140.2–166.3)	157.7 (146.2–184.5)	0.644
After dialysis SBP (mmHg)	155.7 (141.3–169.3)	152.0 (139.6–162.7)	160.7 (143.5–175.7)	0.159
Before dialysis DBP (mmHg)	75.0 (66.6–87.6)	72.3 (66.6–87.5)	80.0 (67.0–90.7)	0.238
After dialysis DBP (mmHg)	80.3 (70.7–87.3)	81.3 (67.2–87.7)	78.7 (66.6–88.8)	0.269
Kt/V	1.4 (1.2–1.7)	1.5 (1.3–1.7)	1.5 (1.3–1.7)	0.402
Water removal amount (L)	2.2 (1.7–3.0)	2.3 (1.7–3.1)	1.5 (1.3–1.7)	0.715
Number of medications	8.0 (5.0–10.0)	7.5 (4.8–9.0)	8.0 (6.0–12.0)	0.006
Laboratory values				
Albumin (g/dL)	3.6 (3.3–3.8)	3.6 (3.4–3.8)	3.6 (3.3–3.8)	0.062
Hemoglobin (g/dL)	11. 0 (10.3–11.7)	11. 0 (10.3–11.7)	11. 0 (10.2–11.6)	0.129
Parathyroid hormone (pg/mL)	147.0 (59.5–312.0)	137.0 (52.0–277.5)	164.0 (87.0–313.5)	0.481
J-CHS (score)	2.0 (2.0–4.0)	2.0 (1.8–3.3)	2.0 (1.5–4.0)	< 0.001
History of fall during a year, number of participants (%)	18 (41.9)	2.0 (1.0)	16.0 (76.1)	0.001
Balance function				
Pre-HD length of CoP (mm)	2165.9 (1756.8–670.3)	1762.2 (1529.8–998.6)	2670.3 (2479.5–2918.6)	0.068
Post-HD length of CoP (mm)	1992.2 (1703.0–2315.1)	1717.8 (1484.9–1816.7)	2315.1 (2115.2–2818.0)	0.025
Pre-HD TUG (s)	8.9 (7.6–10.6)	7.8 (6.9–8.4)	10.6 (9.7–14.7)	< 0.001
Post-HD TUG (s)	9.3 (7.8–11.8)	7.9 (7.0–8.8)	11.8 (10.2–15.0)	< 0.001
Number of faller (%)	24 (55.8)	10.0 (45.5)	14.0 (66.7)	0.012

*BMI* body mass index, *CCI* Charlson comorbidity index, *CoP* center of pressure, *DBP* diastolic blood pressure, *DW* dry weight, *PTH* parathyroid hormone, *SBP* systolic blood pressure, *TUG* timed-up-and-go test

It is critical to intervene in balance disorders to reduce the incidence of falls in HD patients. Balance training included in endurance resistance programs has been shown to improve postural balance in HD patients [31]. It has been reported that dynamic balance training improved TUG, quality of life, and physical function in the elderly women underwent total knee arthroplasty [32]. Together with our results, the intervention in dynamic balance function might reduce fall incidence, possibly improving total physical functions in HD patients.

This study has several limitations. This study comprised a small sample size at a single center. Despite these limitations, to the best of our knowledge, this is the first prospective cohort study to determine the association between balance faction and fall incidence in HD patients. Further studies are needed to confirm our results with large-scale and multicenter studies.

## Conclusions

Frequent accidental falls were observed in patients undergoing HD. Dynamic balance, but not static balance, was associated with the frequency of accidental falls. Evaluation and intervention in balance function might reduce the risk of accidental falls in HD patients.

## Supplementary Information

**Additional file 1.**

**Additional file 2.**

**Additional file 3.**

## Data Availability

The analysis dataset for the current study is available from the corresponding author upon reasonable request.

## Abbreviations

CCI: Charlson Comorbidity Index
CKD: chronic kidney disease
CoP: center of pressure
HD: hemodialysis
TUG: timed up and go test
