# Supplementary material for: Dysfunction in dynamic, but not static balance is associated with risk of accidental falls in hemodialysis patients: a prospective cohort study
Source: BMC Nephrol. 2022 Jul 6;23:237. doi: 10.1186/s12882-022-02877-6 (PMC9260986; doi:10.1186/s12882-022-02877-6)
Supplement: Supplementary file 1 — Additional file 1. [file 12882_2022_2877_MOESM1_ESM.docx]

**Supplement table1. Participants’ clinical characteristics and comparison by Pre-HD length of CoP**

| **Parameters** | **All**  **(n=43)** | **Length of CoP shorter group (n=22)** | **length of CoP longer group (n=21)** | ***p*-value** |
| --- | --- | --- | --- | --- |
| Age (years) | 74.0 (66.0–79.0) | 70.0 (64.8–78.3) | 76.0 (70.0–82.5) | 0.180 |
| Men, n (%) | 20 (46.5) | 11 (50.0) | 9 (42.9) | 0.763 |
| Height (m) | 1.56 (1.47–1.63) | 1.56 (1.47–1.66) | 1.53 (1.48–1.62) | 0.436 |
| DW (kg) | 51.0 (42.8–60.6) | 57.3 (43.9–61.1) | 46.4 (42.5–59.8) | 0.243 |
| Change of DW (%) | 0.0 (-5.0–3.0) | 0.0 (-1.4–1.8) | -0.6 (-5.2–2.3) | 0.715 |
| BMI (DW/m²) | 20.6 (19.0–23.4) | 21.8 (18.9–24.9) | 20.5 (18.8–23.3) | 0.356 |
| Dialysis duration (years) | 5.0 (2.0–11.0) | 4.0 (2.0–9.5) | 9.0 (3.0–13.0) | 0.217 |
| Comorbid conditions |  |  |  |  |
| Cerebrovascular disease, n (%) | 11 (25.6) | 7 (31.8) | 4 (19.0) | 0.488 |
| Cardiac disease, n (%) | 28 (65.1) | 16 (72.7) | 12 (57.1) | 0.347 |
| Diabetes mellitus, n (%) | 19 (44.1) | 10 (45.5) | 9 (42.9) | 1.0 |
| Diabetic retinopathy, n (%) | 9 (20.9) | 3 (13.6) | 6 (28.6) | 0.281 |
| Primary kidney disease |  |  |  |  |
| Diabetic nephropathy, n (%) | 11 (25.6) | 4 (18.2) | 7 (33.3) | 0.310 |
| Glomerulonephritis, n (%) | 14 (32.6) | 6 (27.3) | 8 (38.1) | 0.526 |
| Hypertension, n (%) | 11 (25.6) | 8 (36.4) | 3 (14.3) | 0.162 |
| Other nephropathies, n (%) | 7 (16.3) | 4 (18.2) | 3 (14.3) | 1.0 |
| CCI (score) | 6.0 (4.0–7.0) | 5.5 (4.0–7.0) | 7.0 (5.0–7.0) | 0.178 |
| Blood pressure |  |  |  |  |
| Before dialysis SBP (mmHg) | 154.0 (141.0–172.6) | 153.0 (140.2–163.3) | 161.7 (146.2–188.3) | 0.103 |
| After dialysis SBP (mmHg) | 155.7 (141.3–169.3) | 152.0 (139.6–162.7) | 160.7 (143.5–177.7) | 0.174 |
| Before dialysis DBP (mmHg) | 75.0 (66.6–87.6) | 73.7 (66.8–87.5) | 77.0 (66.5–90.7) | 0.932 |
| After dialysis DBP (mmHg) | 80.3 (70.7–87.3) | 81.3 (67.2–87.7) | 77.3 (68.5–88.8) | 0.593 |
| Kt/V | 1.4 (1.2–1.7) | 1.4 (1.3–1.7) | 1.4 (1.2–1.7) | 0.932 |
| Water removal amount (L) | 2.2 (1.7–3.0) | 2.5 (1.8–3.1) | 1.5 (1.3–1.7) | 0.466 |
| Number of medications | 8.0 (5.0–10.0) | 7.5 (4.8–10.0) | 8.0 (7.0–12.0) | 0.420 |
| Laboratory values |  |  |  |  |
| Albumin (g/dL) | 3.6 (3.3–3.8) | 3.6 (3.4–3.9) | 3.6 (3.3–3.8) | 0.244 |
| Hemoglobin (g/dL) | 11. 0 (10.3–11.7) | 11.2 (10.6–11.6) | 11. 0 (10.1–11.8) | 0.504 |
| Parathyroid hormone (pg/mL) | 147.0 (59.5–312.0) | 123.0 (52.0–273.0) | 185.0 (87.0–320.0) | 0.296 |
| J-CHS (score) | 2.0 (2.0–4.0) | 2.0 (1.8–3.3) | 2.0 (1.5–4.0) | 0.802 |
| History of fall during a year, number of participants (%) | 18 (41.9) | 7 (31.8) | 11 (52.4) | 0.750 |
| Balance function |  |  |  |  |
| Pre-HD length of CoP (mm) | 2165.9 (1756.8–2670.3) | 1762.2 (1529.8–1923.4) | 2670.3 (2480.4–2918.6) | <0.001 |
| Post-HD length of CoP (mm) | 1992.2 (1703.0–2315.1) | 1733.4 (1484.9–1940.1) | 2312.7 (2050.8–2818.0) | <0.001 |
| Pre-HD TUG (s) | 8.9 (7.6–10.6) | 8.6 (7.4–9.6) | 9.0 (7.5–10.2) | 0.141 |
| Post-HD TUG (s) | 9.3 (7.8–11.8) | 9.3 (7.8–9.3) | 9.8 (8.7–12.2) | 0.148 |
| Number of faller (%) | 24 (55.8) | 10.0 (45.5) | 14 (66.7) | 0.521 |

*BMI* body mass index, *CCI* Charlson comorbidity index, *CoP* center of pressure, *DBP* diastolic blood pressure, *DW* dry weight, *PTH* parathyroid hormone, *SBP* systolic blood pressure, *TUG* timed-up-and-go test.
