# Supplementary material for: Dysfunction in dynamic, but not static balance is associated with risk of accidental falls in hemodialysis patients: a prospective cohort study
Source: BMC Nephrol. 2022 Jul 6;23:237. doi: 10.1186/s12882-022-02877-6 (PMC9260986; doi:10.1186/s12882-022-02877-6)
Supplement: Supplementary file 2 — Additional file 2. [file 12882_2022_2877_MOESM2_ESM.docx]

**Supplementa**
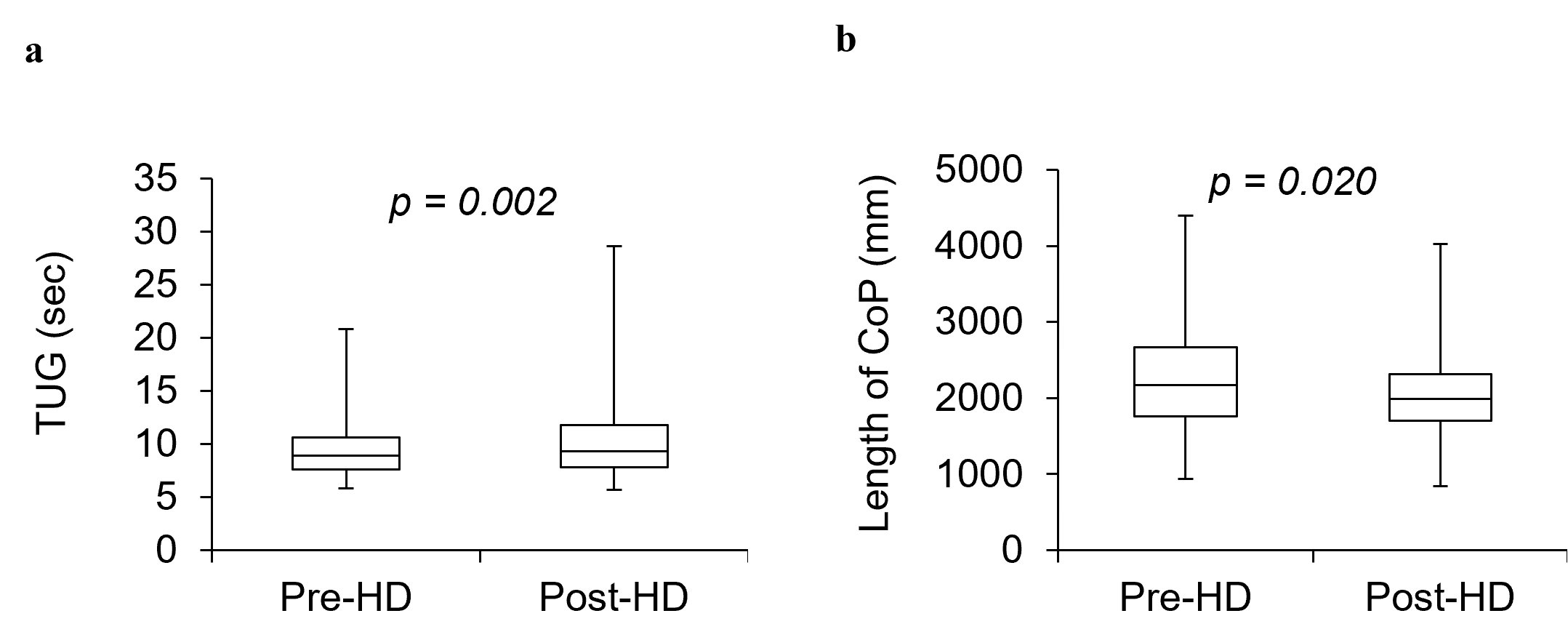
**l figure 1.** Comparison of balance functions between pre- and post-hemodialysis (HD) sessions. **a** Timed-up-and-go test (TUG) and **b** length of center of pressure (CoP) are shown.
