# Supplementary material for: Dysfunction in dynamic, but not static balance is associated with risk of accidental falls in hemodialysis patients: a prospective cohort study
Source: BMC Nephrol. 2022 Jul 6;23:237. doi: 10.1186/s12882-022-02877-6 (PMC9260986; doi:10.1186/s12882-022-02877-6)
Supplement: Supplementary file 3 — Additional file 3. [file 12882_2022_2877_MOESM3_ESM.docx]

**Supplement table2. Relationship between the number of falls and balance functions adjusted with age, history of falls and number of medications**

|  | Unadjusted model | | Adjusted model^*^ | |
| --- | --- | --- | --- | --- |
|  | B (95% CI) | *p*-value | B (95% CI) | *p*-value |
| Pre-HD TUG | 0.297 (0.190, 0.404) | <0.001 | 0.219 (0.079, 0.360) | 0.003^a^ |
| Post-HD TUG | 0.266 (0.189, 0.344) | <0.001 | 0.219 (0.117, 0.320) | <0.001^b^ |
| Pre-HD length of CoP | 0.001 (0.000, 0.002) | 0.006 | 0.000 (0.000, 0.001) | 0.281^c^ |
| Post-HD length of CoP | 0.001 (0.000, 0.001) | 0.033 | 0.001 (-0.001, 0.001) | 0.764^d^ |

CI: Confidence Interval, CoP: Center of Pressure, TUG: Timed-up-and-go test

^*^Adjusted model: Age, number of medications, history of fall during a year

^a^Adjusted R2 = 0.438, ^b^adjusted R2 = 0.527, ^c^adjusted R2 = 0.312, and ^d^adjusted R2 = 0.292
